# Serial MRI studies over 12 months using manual and atlas-based region of interest in patients with amyotrophic lateral sclerosis

**DOI:** 10.1186/s12880-020-00489-w

**Published:** 2020-08-03

**Authors:** Ashwag R. Alruwaili, Kerstin Pannek, Robert D. Henderson, Marcus Gray, Nyoman D. Kurniawan, Pamela A. McCombe

**Affiliations:** 1 The University of Queensland, Centre for Clinical Research, Brisbane, Australia; 2 King Saud University, Riyadh, Saudi Arabia; 3 School of Medicine, The University of Queensland, Brisbane, QLD Australia; 4 Australian E–Health Research Centre, CSIRO, Brisbane, Australia; 5 The Department of Neurology, Royal Brisbane and Women’s Hospital, Herston, Brisbane Australia; 6 Centre for Advanced Imaging, The University of Queensland, Brisbane, Australia; 7 Gehrmann Laboratory, University of Queensland, Brisbane, QLD Australia

**Keywords:** Amyotrophic lateral sclerosis, ALS, Motor neuron disease, MND, Diffusion tensor imaging, DTI, Region of interest, ROI, Tract-based spatial statistics, TBSS

## Abstract

**Background:**

Amyotrophic lateral sclerosis (ALS) is a neurodegenerative disease characterized by loss of upper and lower motor neurons. There is a need for an imaging biomarker to track disease progression. Previously, magnetic resonance imaging (MRI) has shown loss of grey and white matter in the brain of patients with ALS compared to controls. We performed serial diffusion tractography imaging (DTI) study of patients with ALS looking for changes over time.

**Methods:**

On all subjects (*n* = 15), we performed three MRI studies at 6 month intervals. DTI changes were assessed with tract-based spatial statistics (TBSS) and region of interest (ROI) studies. Cortic-spinal tract (CST) was selected for our ROI at the upper level; the posterior limb of internal capsule (PLIC), and a lower level in the pons.

**Results:**

There was no significant change in DTI measures over 12 months of observation. Better correlation of manual and atlas-based ROI methods was found in the posterior limb of the internal capsule than the pons.

**Conclusion:**

While previous DTI studies showed significant differences between ALS subjects and controls, within individual subjects there is little evidence of progression over 12 months. This suggests that DTI is not a suitable biomarker to assess disease progression in ALS.

## Background

Amyotrophic lateral sclerosis (ALS) is characterized by the progressive loss of lower motor neurons (LMN) from the spinal cord and brain stem and upper motor neurons (UMN) from the motor and premotor cortex. There can also be extra-motor involvement [1]. The pathology of ALS includes aggregation of protein within cells [2]. The protein accumulation spreads to other regions as disease progresses [3]. There is a need for non-invasive biomarkers as a means of monitoring progression of patients with ALS, for prognosis and for use in clinical trials. Possible biomarkers include clinical, neurophysiological, blood and imaging measurements. An imaging biomarker is desirable because it can show changes in brain structure that can be related to the pathology of disease.

Magnetic resonance imaging (MRI) has an advantage of being non-invasive, and able to quantify brain structure and to make inferences about tissue pathology. Cross sectional MRI studies in ALS have shown abnormalities in voxel–based morphometry (VBM) and diffusion weighted imaging (DWI) [4–7]. Because ALS is a disease that shows degeneration of white matter fiber tracts, particularly the corticospinal tract (CST), there have been studies that use diffusion tensor imaging (DTI). DTI can be analyzed with Tract-Based Spatial Statistics (TBSS) [8] or region of interest (ROI) studies, which can be can be performed by manual or semi-automated methods. Manually drawn ROIs have been considered to have problems with reproducibility, but have the advantage of ease of use.

All DTI metrics have been found to reflect microstructural pathology. What we know about DTI metrics is largely based upon empirical studies that investigate how these measures correlate with the underlying pathology. Quantitative FA measures are believed to reflect changes in the myelination, fiber density and packing [9]. Evidence suggests that fractional anisotropy (FA) is sensitive to the WM integrity but is not very specific and cannot distinguish between different pathological processes [10–12]. Mean diffusivity (MD) is suggested to be influenced by free water diffusion in cellular space, such as oedema, which is increased in neurodegenerative diseases [12]. Axial diffusivity (AD) is thought to reflect demyelination in white matter tracts. Axial integrity of axons and can be reflected as either increased or decreased AD when WM is destroyed by any other process [13]. It has not yet been well-defined whether radial diffusivity (RD) is specific to demyelination and could also be related to be due to; the occurrence of inflammation, or to axonal damage. RD was found to be high in neurodegenerative disease [14]. RD has been shown to correlate with demyelination and dysmyelination in several pathological models [15, 16].

Cross-sectional DTI studies have consistently shown reduction in FA in the corticospinal tract (CST) in ALS patients compared to controls [17–20]. In contrast, longitudinal DTI studies of ALS have shown conflicting findings, but most studies show DTI abnormalities early in disease, but little progression over time [7, 21–26].

We have previously reported a study comparing scans 6 months apart, and found little difference in DTI measures [24]. In this study we have focused on the CST, because degeneration of the CST is a cardinal feature of the pathology in ALS. To examine whether changes can be detected after a longer interval, we now report a 12 month serial study of patients with ALS, with the aim of determining whether there are changes over time and the second aim was to compare semi-automated ROI with manual ROI methods.

## Methods

### Subjects

Patients (*n* = 15) with clinically definite or clinically probable ALS, according to the revised El Escorial criteria [27] were recruited from the motor neuron disease (MND) clinic of the Royal Brisbane and Women’s Hospital (RBWH) and were included in previous studies [20, 24]. They had MRI imaging three times at 6 monthly intervals. Disease severity was measured using the revised ALS Functional Rating Scale (ALSFRS-R) [28]. This project was approved by the RBWH ethics committee HREC (2008/98). All subjects provided written informed consent.

### MRI acquisition

All MRI scans were performed at the Royal Brisbane and Women Hospital (RBWH) using a 3 T Siemens Tim Trio (Siemens, Erlangen, Germany) with a 12-channel parallel head coil. Diffusion-weighted images (DWIs) were acquired along 64 non-collinear directions at b = 3000 s/mm^2^, with one non-diffusion weighted image. Acquisition parameters were: 60 axial slices, FOV 30 × 30 cm, slice thickness 2.5 mm, matrix 128 × 128, TR/TE 9700/119 ms, iPAT factor 2. A field map was acquired using two 2D gradient-recalled echo images with TE1/TE2 = 4.92/7.38 ms and TR = 488 ms to assist in the correction of geometric distortions. The acquisition time for the diffusion dataset was 9:40 min.

In addition, a high-resolution structural image was acquired using a 3D *T*_*1*_ MPRAGE (FOV 23 cm slice thickness 0.9, 0.44 × 0.44 mm in-plane resolution, matrix 24 × 25.6 × 17.6 cm, TR/TE/TI 1900/2.4/900 ms, flip angle = 9 degrees).

### Diffusion processing

Diffusion MRI data were preprocessed as described previously [29]. Preprocessing included correction for head movement with rotation of the b-matrix, detection and removal of signal intensity outliers, and correction for geometric distortions and intensity inhomogeneity. Maps of FA were calculated using MRTrix version 0.2.9 [30].

### TBSS analysis

Tract-based spatial statistics (TBSS) is a tool for analyzing DTI variables, which allows objective voxel-wise analysis. TBSS provides a powerful method for detecting variation on major white matter tract. The alignment of the DT images to a reference space is required. This allows spatially overlapping voxels of different datasets to correspond to the same anatomical structures.

Following registration, the mean FA-image was created and thinned to represent the mean FA skeleton. Individual FA volumes were projected onto this common skeleton [31]. Next, data were fed into voxel-wise cross-subject statistical analysis with different group comparisons, including a paired test. The statistical analyses employed the voxel-wise general linear model (GLM) and significant clusters were formed by employing the TFCE method to correct for multiple comparisons [32], implemented in FSL randomize [33]. *P*-values were determined using 5000 random permutations [33]. The results are considered significant at *p* < 0.05, corrected for multiple comparisons using TFCE.

To analyze differences among three groups, we combined all the scans and ran the TBSS steps as above to create FA skeleton of whole group. Before the randomise step, we separated scans for each time-point from the FA skeleton. Next, two time-points were merged to one skeleton. Then, using randomise with multiple permutations and correcting for multiple comparisons using threshold-free cluster enhancement (TFCE, [32]), which is an approach to avoid the problem of thresholding selection in the context of cluster-based statistics. At *p* < 0.05, we ran two different statistical approaches: (a) one sample t-test; by subtracting each time-point from the other one in six contrasts (1 minus 2, 1 minus 3, 2 minus 1, 2 minus 3, 3 minus 1, 3 minus 2) and the output is the difference between time-points; (b) paired sample t-test; comparing two time-points each time, separately, using the contrast as (scan 1 versus scan 2, scan 2 versus scan 3, scan 1 versus scan 3); c) triple t-test; comparing all three time-points. The final results from TBSS comparisons of ALS patients at each time-point were superimposed using FSL (http://fsl.fmrib.ox.ac.uk/fsl/fslwiki/).

### Automated ROI analysis methods

For the automated ROI, Advanced Normalization Tools (ANTs) was performed on FA data using the methods described [24]. A custom FA template was derived from all subjects, using scripts provided with ANTs (http://picsl.upenn.edu/ANTS/) [34]. We used ANTs symmetric diffeomeophic registrations using symmetric image normalization (Greedy SyN). The JHU 1-mm FA was used for the initial rigid body registration to generate the template.

The JHU atlas [35] was normalized to this study template using symmetric diffeomorphic registration. ROIs of the JHU atlas were subsequently transformed to the individual datasets in native space and mean values of FA were calculated for each ROI.

The datasets were analysed by one blinded rater (A,R A). Manual ROI were drawn using FSL (http://www.fmrib.ox.ac.uk/fsl/) on the basis of the prior anatomic knowledge and relevant literature and saved as a mask [36].

**Fig. 1:**
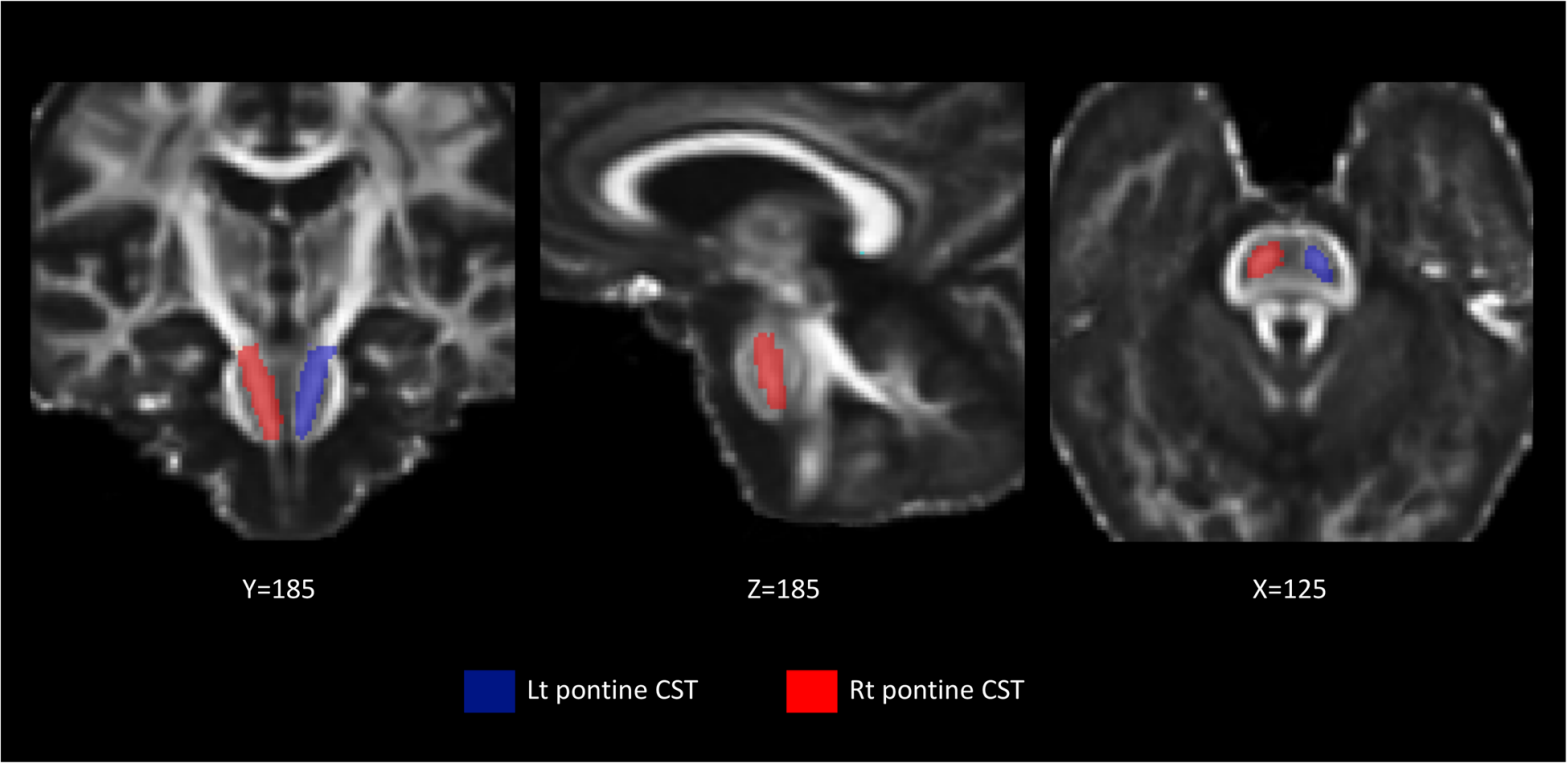
Atlas-based ROI of the CST at the pons. This Figure illustrates, in 3 planes (X, Y and Z), the mask applied for the CST in the pons using the FA template with atlas-based ROI. (Left in blue and right in red). With this method, all the voxels from the entire structure are included in the ROI

**Fig. 2:**
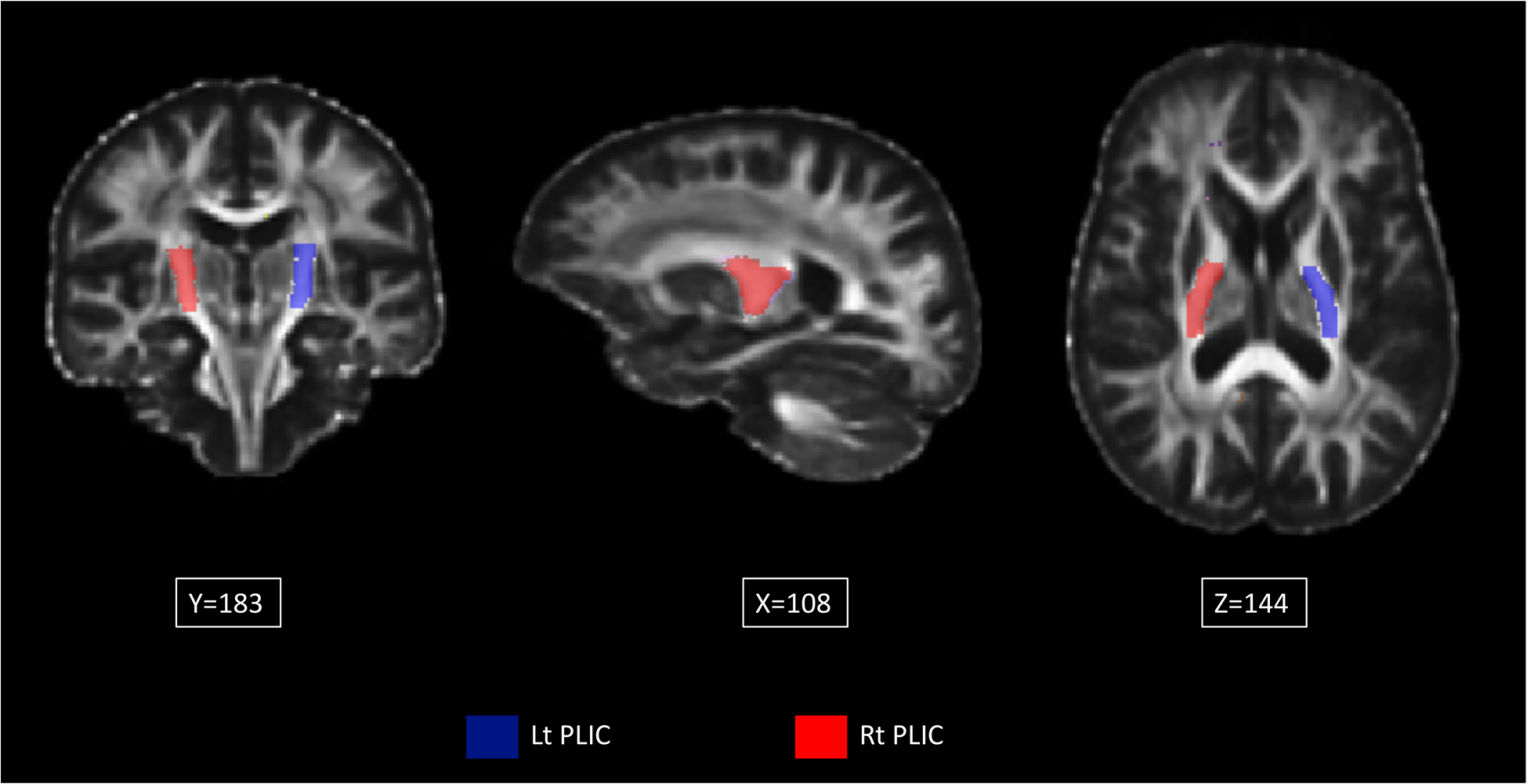
Atlas-based ROI of the PLIC. This Figure illustrates, in 3 planes (X, Y and Z), the mask applied for the PLIC, using the FA template with atlas-based ROI. (left in blue and right in red). With this method, all the voxels from the entire structure are included in the ROI

We examined the CST at the level of pons as shown in Fig. 1 and the posterior limb of the internal capsule (PLIC) as shown in Fig. 2. These regions were chosen because degeneration of the CST is a cardinal feature of ALS. PLIC and pons are part of the CST where PLIC is upper and pons is lower CST. We included the PLIC, the nearest CST to Gray matter (GM), expecting the highest sensitivity where highest neuronal alignment and no crossing fibers present. The FA values were calculated pixel-by-pixel, and average values for each region on both sides were tabulated for statistical analysis.

### Manual ROI methods

**Fig. 3:**
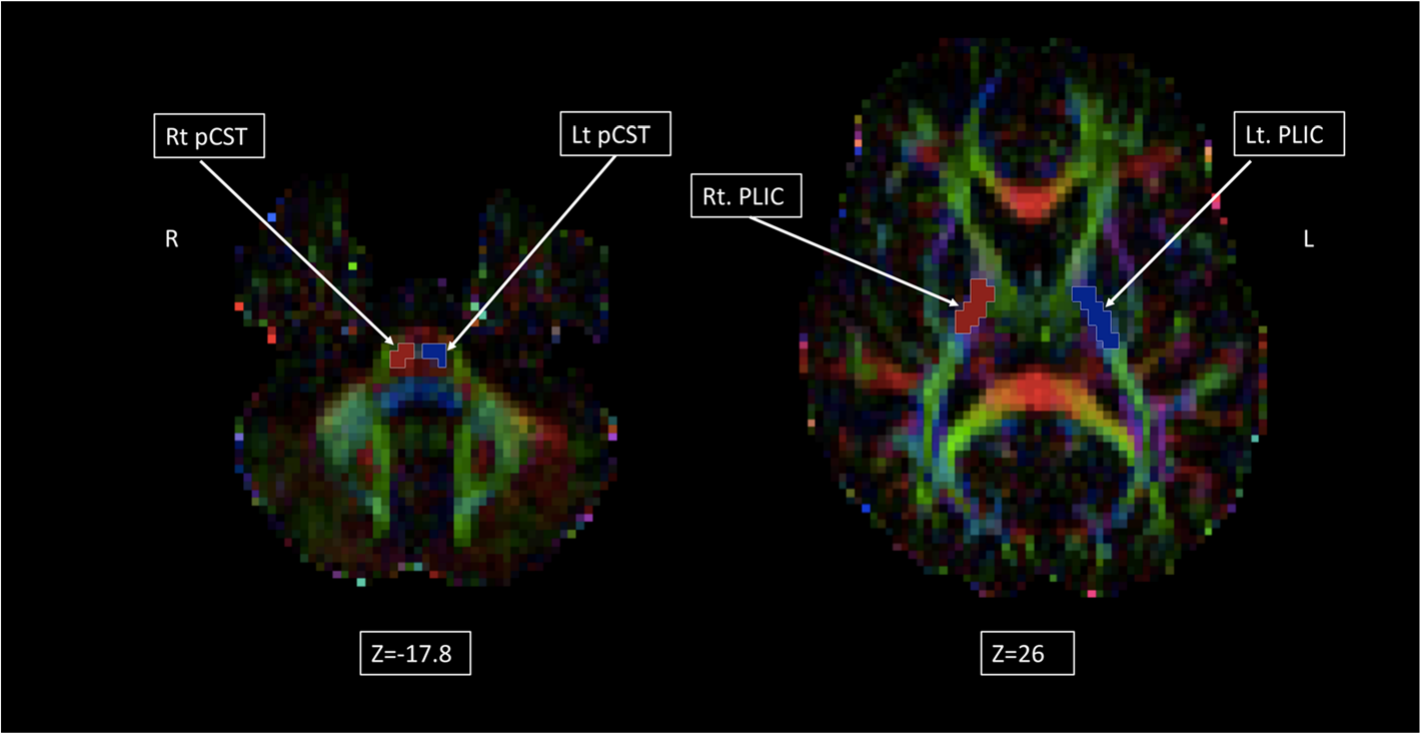
Manual ROI of the CST at the pons and PLIC. This Figure illustrates in two different axial planes for individual patient FA maps and the mask applied for the manual ROI for the pontine CST (left) and posterior limb of internal capsule, PLIC (right). With this method, only the voxels from one plane are included in the mask of the ROI

Using knowledge of anatomy, manual ROIs were drawn on the individual colored FA maps. One axial slice was selected for each region. To delineate the CST in the pons, the ROI was drawn where the pontine crossing tracts (pct) are a straight red line. The pontine CST can be seen as two blue-purple regions anterior to the pct (Fig. 3, left). For the PLIC, the ROI was drawn on the last axial slice where the genu and splenium of corpus callosum and the fornix are seen in one plane. On colored FA maps, the PLIC appears as a blue-purple region; anterior to it is the anterior limb of internal capsule (ALIC) and posterior to the PLIC is the retrolenticular part of the internal capsule (rLIC), (Fig. 3, right). To assess repeatability of ROIs, five patients were randomly selected to have a repeat of the manual ROI, with the repeat analysis done a minimum of 1 month after the first analysis.

### Statistical analysis

Statistical analyses were performed using SPSS for Mac (Ver. 23.0, SPSS Inc., Chicago, IL, USA). Mean and standard deviation values were calculated for each variable. All data were tested for normality using Shapiro–Wilk test. MANOVA was performed when testing ROIs at three time-points for each region of interest. We pooled all the FA data from all subjects from all time-points to examine the overall trend of the relationship between both ROI methods. We examined the relationship between manual ROI and atlas ROI using Spearman’s correlation. Repeatability of the manual ROI was calculated using within-subject coefficient of variation (CV) and the intra-class correlation coefficient (ICC).

## Results

### Subjects

Fifteen ALS patients (mean age 60 ± 13, 10 males and 5 females) were enrolled (Tables 1 and 2). The subjects had a disease duration (DD), that was calculated from the date of onset to the first MRI scan, and ranged from 3 to 118 months. Physical decline in ALSFRS-R are available in Table 3.

**Table 1:** Summary of subjects demographics

	ALS *N* = 15
Age (years) Mean (SD)	60 ± 13*
Gender (Male:female)	10:5^a^
ALSFRS-R Mean (SD)	38.7 ± 4.5
Disease duration Mean (SD) Median (months)	35 ± 27 29
Range of disease duration (months)	3–118
Number of patients deceased at end of study	6

* *p* = 0.005; ^a^ not significant

**Table 2:** Detailed clinical information of ALS subjects

	ID	Familial/Sporadic	Handedness	RILUZOLE	Site of Onset	Date of Onset	Disease duration
1	303	S	Right	N	LUL	01/05/2010	33
2	304	S	Right	N	RUL	01/08/2008	54
3	306	S	Right	Y	RUL	01/10/2010	29
4	308	S	Right	N	LL (Bilateral)	01/01/2008	61
5	311	S	Right	Y	LUL	01/06/2010	34
6	313	S	Right	Y	RLL	01/05/2011	25
7	315	S	Right	Y	LUL	01/03/2012	15
8	316	F	Right	N	LUL	01/08/2013	3
9	318	S	Right	N	RUL	01/08/2012	14
10	319	S	Right	NA	LLL	01/09/2010	37
11	320	S	Right	N	RUL	01/01/2004	118
12	324	S	Right	N	LLL	01/07/2012	20
13	325	S	Right	N	Bulbar	01/09/2011	31
14	329	S	Left	N	LLL	01/02/2012	29
15	333	S	Right	N	Bulbar	01/07/2013	23

**Table 3:** ALS subjects’ physical decline

Subject ID	First ALSFRS-R	Last ALSFRS-R
1	42	34
2	47	45
3	37	30
4	33	33
5	35	42
6	NA	NA
7	31	29
8	NA	NA
9	38	30
10	34	NA
11	48	42
12	41	40
13	46	41
14	43	42
15	44	NA

### TBSS results

The detailed results of TBSS analyses are shown in Table 4. There was no significant difference among the scans of ALS subjects at three time-points when comparing whole ALS group or when excluding patients with longer disease duration more than 40 months (*n* = 3, *p* < 0.05; corrected TFCE). The changes in the CST and corpus callosum (CC) were more widespread at the third scans than in the earlier time-points.

**Table 4:** Results of comparisons that were examined with TBSS. All group comparisons were not significant

*Comparison of 3 groups*			
Group 1	Group 2	Group 3	Significance
Time-point 1 (*N* = 15)	Time-point 2 (*N* = 15)	Time-point 3(*N* = 15)	NS
Time-point 1 exclude longer DD (*N* = 12)	Time-point 2 excluding longer DD (*N* = 12)	Time-point 3 excluding longer DD (*N* = 12)	NS

*DD* Disease duration, *NS* Not significant

### ROI results

**Fig. 4:**
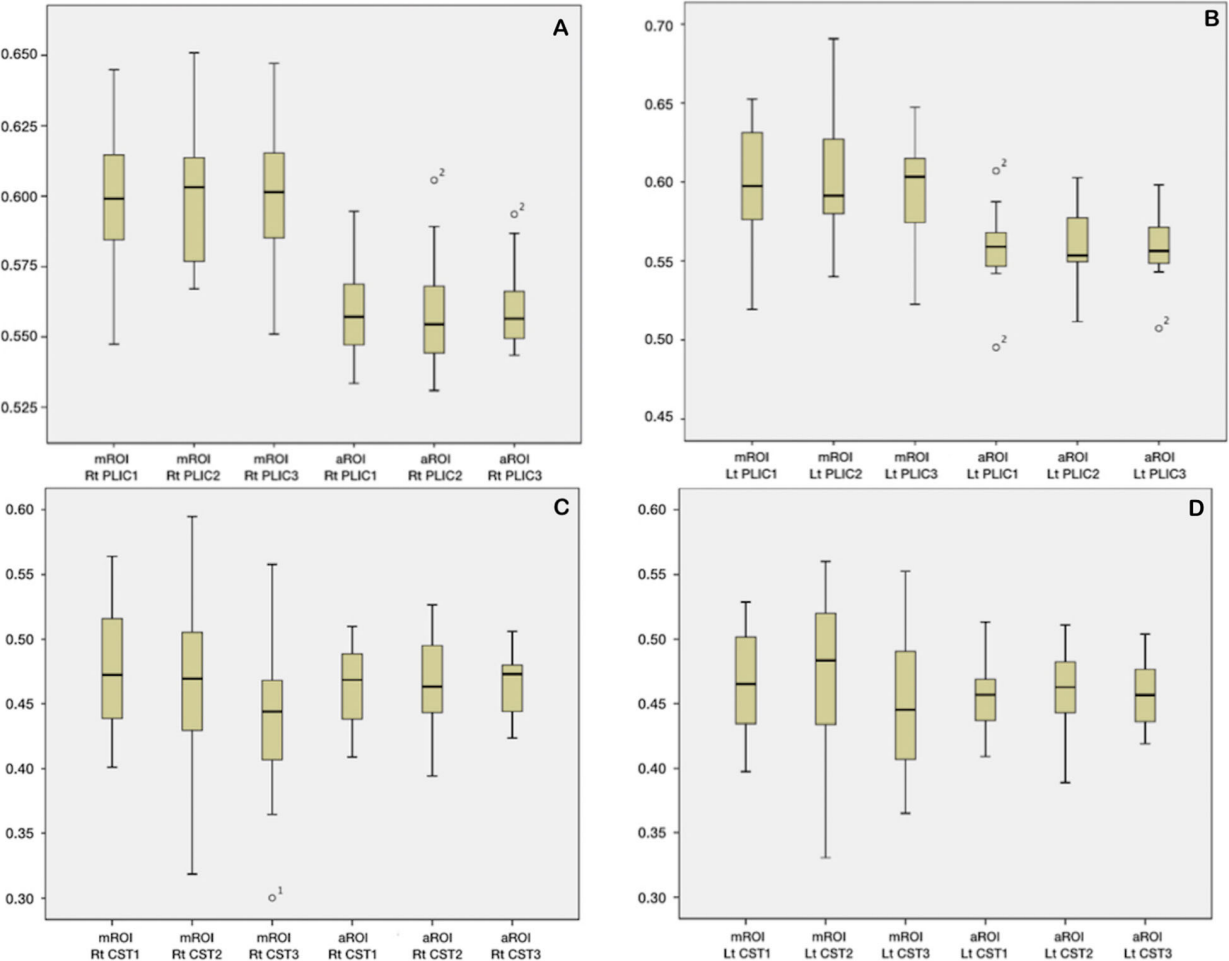
The FA mean and median differences for each ROI in every time-point using both ROI methods. **a** Automated ROI (aROI) in the posterior limb of the right internal capsule (PLIC) show lower FA values than manual ROI (mROI). **b** aROI in the posterior limb of left internal capsule (PLIC) show lower FA values than mROI. **c** Both ROI methods in CST in the right pons show similar results **d** Both ROI methods in CST in the left pons show similar results

With both atlas-based and with manual ROI analysis of FA, there was considerable variation among individuals (Supp Figures 1 and 2). We performed MANOVA to determine whether the average FA values were significantly different over time (Supp Table 1). There was no significant difference among the three time-points for either method. Therefore, we acquired the estimated marginal means for each time-point using both methods to test whether both methods are comparable (Supp Figure 3). There was better agreement for the PLIC than for the CST. A summary of the FA results in all ROIs is shown in Fig. 4.

To compare the FA value between the PLIC and pontine CST, we have combined the 90 FA values for both sides from all time-points and calculated the mean for two methods (Supp Table 2). The average FA from the PLIC is significantly greater than that of the CST in the pons, using both methods.

**Fig. 5:**
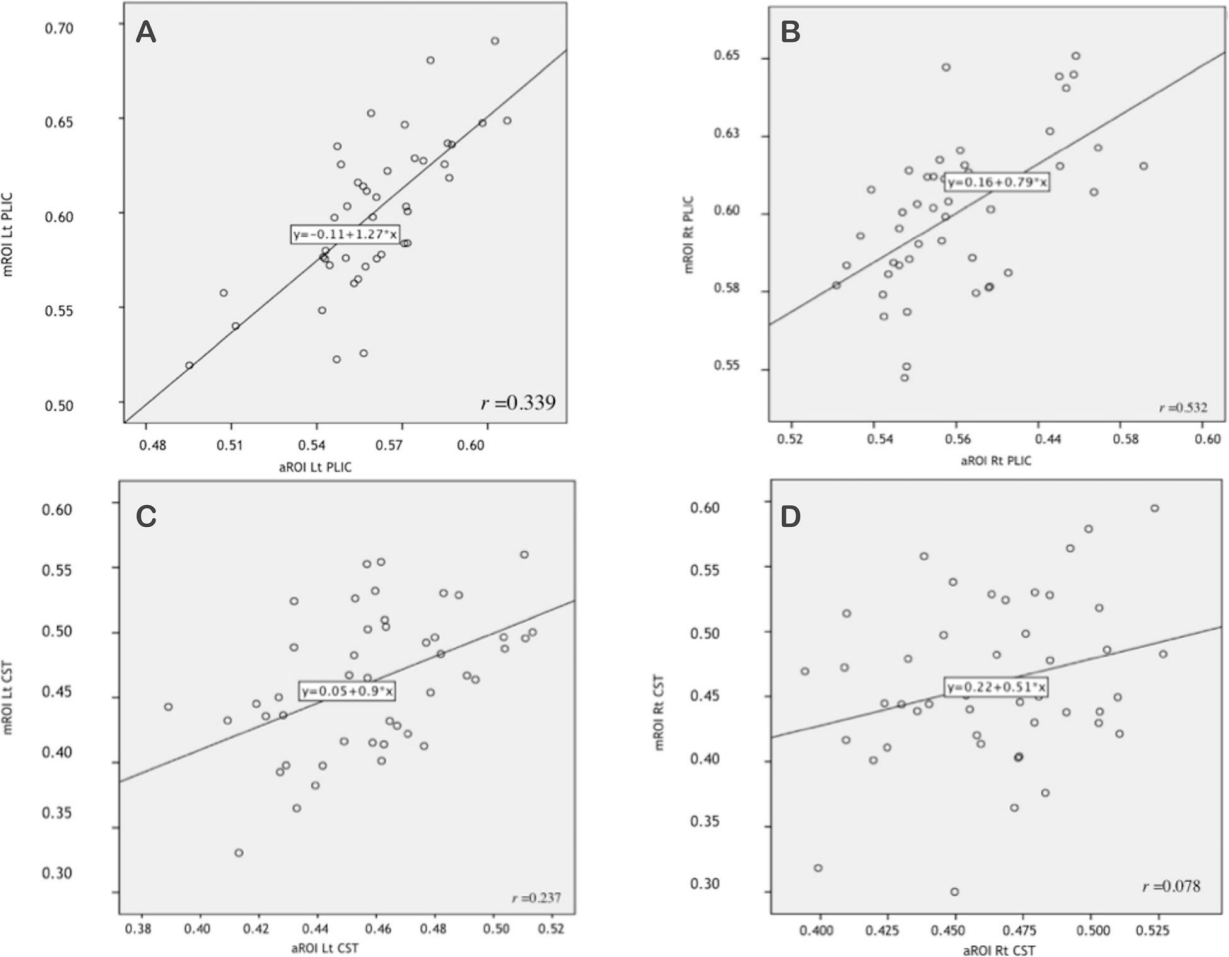
Regression results for FA values for all scans in every ROI comparing manual ROI and automated ROI. **a** Regression analysis of mROI versus aROI for the left PLIC. **b** Regression analysis of mROI versus aROI for the right PLIC. **c** Regression analysis of mROI versus aROI for the left CST in the pons. **d** Regression analysis of mROI versus aROI for the right CST in the pons

A linear regression was performed between atlas-based and manual-based measurements for each ROI using all the observations from the three time-points (Fig. 5). There was better agreement for the values from the PLIC than the values from the pons. We also performed a Spearman’s correlation of ROI measurements to compare the manual versus atlas-based ROI methods testing if both methods are comparable (Supp Table 3). The correlation in the left PLIC was significant (*p* = 0.01, *r* = 0.6).

### Reproducibility of manual ROI

Reproducibility of manual ROI was tested with 1 month difference between manual ROI placements. The within-subject CV was used to test repeatability of both ROI methods. In the right PLIC, CV was 0.1–3.6% in the aROI and was 0.007–11% in the mROI.

In terms of reliability, the inter-rater and intra-rater variability can be assessed using the ICC [37]. This have been reported to be between 17 and 37% [38]. In our study, ICC was between 24 and 38% for the right pons while the left pons was 74–85%. In the right PLIC, the ICC was 37–54% and the left PLIC was 65–79%.

## Discussion

This study shows that there is no significant change in DTI along CST in the selected ROIs in ALS patients over 12 months. Manual and automated ROI methods gave comparable results.

The underlying pathology of ALS is death of upper motor neurons, degeneration of the CST, and death of lower motor neurons, with eventual gliosis of the CST in the spinal cord. In the present study, we have examined changes in DTI measures, with the hypothesis that there would be degeneration over time seen as loss of FA in WM tracts. We used TBSS for an overview of all the fiber tracts and ROI of the PLIC and CST to determine whether MRI can show changes in these motor pathways. We find that there is no significant change in DTI over a 12 month period. We also find that the PLIC shows better correlation between manual and automated ROI measures than pons.

Longitudinal DTI studies in ALS are challenging and the findings are variable, but generally concur that fiber tract degeneration has typically taken place by the time the diagnosis is confirmed, with limited additional WM changes in later phase of the disease [39]. An older longitudinal DTI study with only six noncollinear directions have reported positive findings but on a very small sample size [40]. Our own previous study found little change in DTI over 6 months [24]. In the present study we examined changes over a 12 month period, to see whether DTI changes appear over a longer time interval.

Data were investigated at three different time-points, 6 months apart. With TBSS there was some evidence of change over time in motor pathways. The ROI analyses were used to test our hypothesis that there would be changes in the FA in the CST at pons and PLIC. There were no significant differences in the average FA between time-point using aROI. However, in this study some patients showed loss of FA over time while others did not. This difference between TBSS and ROI has been noted previously, in a study that found ROI methods to show less significant results than TBSS methods [41].

There are some reasons why there would be little change in FA over time. Firstly, ALS patients are heterogeneous, with some patients showing prolonged survival [42–44]. In our cohort, some of the patients had prolonged survival, indicating slowly progressive disease, and therefore would not be expected to show rapid change in fiber tracts. It may be that patients with rapidly progressing disease would show DTI changes.

Next, it is likely that after a fiber tract has completely degenerated, there will be no further change in DTI. Some of our patients were in later stages of the disease, when fiber tracts could be completely degenerated. If loss of fiber tracts leads to decline in FA, then the decline would be expected to cease at this point.

However, it is also possible that, even early in disease, the degeneration in the motor tracts is substantially advanced. Other studies have reported little change in DTI over time [7, 45]. These authors suggested that this indicates that upper motor neuron degeneration occurs early in the disease. Our previous MRI study showed that UMN degeneration occurs early [46], and neurophysiological studies also suggest this [47]. This could mean that changes in FA occur early in disease. It may be that it is better to measure spread of DTI changes to new regions rather than follow changes in the CST [48]. Heterogeneity of clinical features in ALS patients is also an issue and subgroup analysis according to features such as the presence of cognitive impairment would be useful.

In the current study we compared atlas based and manual ROI. The manual methods gave a wider spread of values of FA. We found better agreement from the PLIC than the CST in the pons. Linear regression showed a significant relationship between the manual and atlas based methods for the PLIC but not the pons. This can be explained by the crossing WM pathways in the pons which can lead to a substantial drop of FA in the map. Using Spearman’s correlation, there was a significant correlation of the values for the left PLIC. Manual ROI has been criticized for being operator dependent [49]. However, we found that both methods are still comparable which is in support of previous studies [50, 51]. Rigorous published work have compared different automated methods [52] and test-retest reproducibility [53] in segmentation approaches suggests that atlas-based methods are the best [54].

Laterality was not the aim in the current study. However, it is possible that our lack of significant difference in pons was due to not only the presence of crossing fibers but also to the challenges in delineating this structure, due to its small size, and difficulty discerning its boundaries within the brain stem. However, this variability in pons does limit the interpretation of our findings.

Previous studies have reported that although inter-site DTI measurement variability was found to be quite high [55], the intra-subject variation was less than 1% in different brain regions using DTI [56]. Our patients showed variation of more than 1%, therefore changes we observed are unlikely to be caused by measurement error alone. Compared to previous studies, the ICC from the current study shows that the manual ROI has a good reproducibility and the regions were drawn consistently [57].

Limitations of our study include the small sample size. More investigations are needed to characterize different ALS stages with a larger group of cohorts.

## Conclusion

In conclusion, we found little evidence of progression of ALS using DTI. It may be that there is little further change after diagnosis because the pathological changes are complete. This study adds more information about the use of DTI in ALS longitudinal studies.

## Supplementary information

**Additional file 1 Figure S1**. Atlas-based ROI FA measurement for individual patients at three time- points in the PLIC and CST in the pons. Using MANOVA there were no significant difference among the mean values at each time-points.

**Additional file 2 Figure S2**. Manual ROI FA measurement for individual patients at three time-points for the CST at the pons and PLIC. Using MANOVA there were no significant difference among the mean values at each time-points.

**Additional file 3 Figure S3**. Estimated marginal means for every ROI in both methods (mROI in blue (1) and aROI in green (2)). There was better agreement for the PLIC (upper panels) than for the CST in the pons (lower panels).

**Additional file 4 Table S1**. Means (SD) for FA at three Time-points using atlas-based ROI and manual ROI. None of the regions, PLIC and CST at pons, showed significant differences overtime.

**Additional file 5 Table S2.** FA averaged mean of ROIs in both methods.

**Additional file 6 Table S3.** Correlation of FA in both methods. Left PLIC showed significant correlation using both ROI methods.

## Data Availability

The datasets generated and analysed during the current study are not publicly available but are available from the principal investigator Prof. Pamela McCombe pamela.mccombe@uq.edu.au on reasonable request.

## Abbreviations

ALS: Amyotrophic lateral sclerosis
ALSFRS-R: The revised ALS Functional Rating Scale
ALIC: Anterior limb of internal capsule
aROI: Automated Region of interest
AD: Axial diffusivity
CC: Corpus callosum
CST: Corticospinal tract
CV: Coefficient of variation
DTI: Diffusion tensor imaging
DWI: Diffusion weighted imaging
DD: Disease duration
FA: Fractional anisotropy
FOV: Field of view
GLM: General linear model
GM: Gray matter
ICC: Intraclass correlation coefficient
LL: Left limb
LMN: Lower motor neuron
LUL: Left upper limb
MD: Mean diffusivity
mROI: Manual region of interest
MND: Motor neuron disease
MRI: Magnetic resonance imaging
NS: Not significant
PLIC: Posterior limb of internal capsule
pct: Pontine crossing tracts
RD: Radial diffusivity
rLIC: Retrolenticular part of the internal capsule
TBSS: Tract-based spatial statistics
TE: Echo time
TFCE: Threshold-free cluster enhancement
TI: Inversion time
TR: Repetition time
UMN: Upper motor neuron
ROI: Region of interest
rLIC: Retrolenticular part of internal capsule
RUL: Right upper limb
VBM: Voxel–based morphometry
WM: White matter
