# Supplementary figures and images for: Serial MRI studies over 12 months using manual and atlas-based region of interest in patients with amyotrophic lateral sclerosis
Source: BMC Med Imaging. 2020 Aug 3;20:90. doi: 10.1186/s12880-020-00489-w (PMC7397614; doi:10.1186/s12880-020-00489-w)

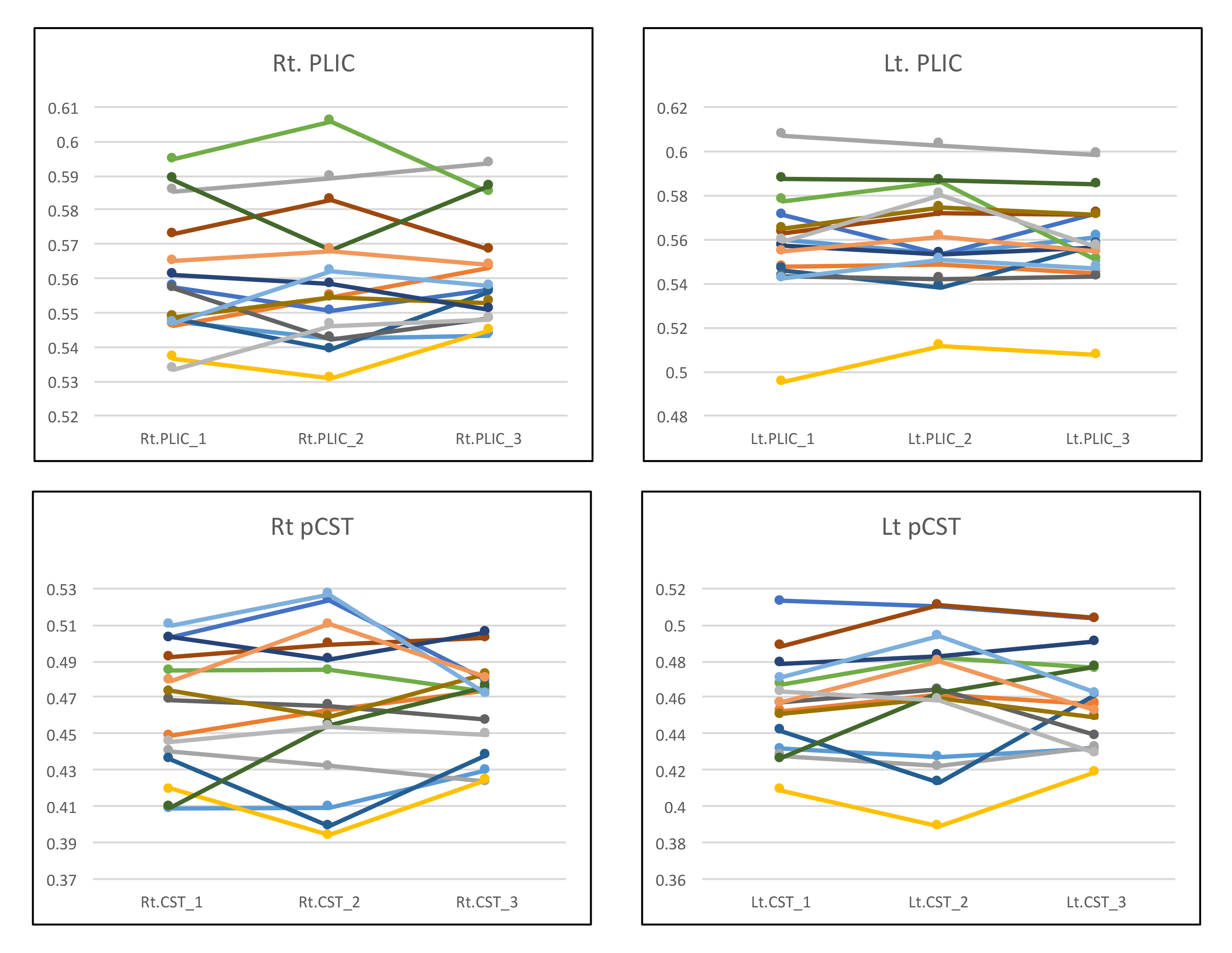

Supplement: Supplementary file 1 — Additional file 1 Figure S1. Atlas-based ROI FA measurement for individual patients at three time- points in the PLIC and CST in the pons. Using MANOVA there were no significant difference among the mean values at each time-points. [file 12880_2020_489_MOESM1_ESM.tif]

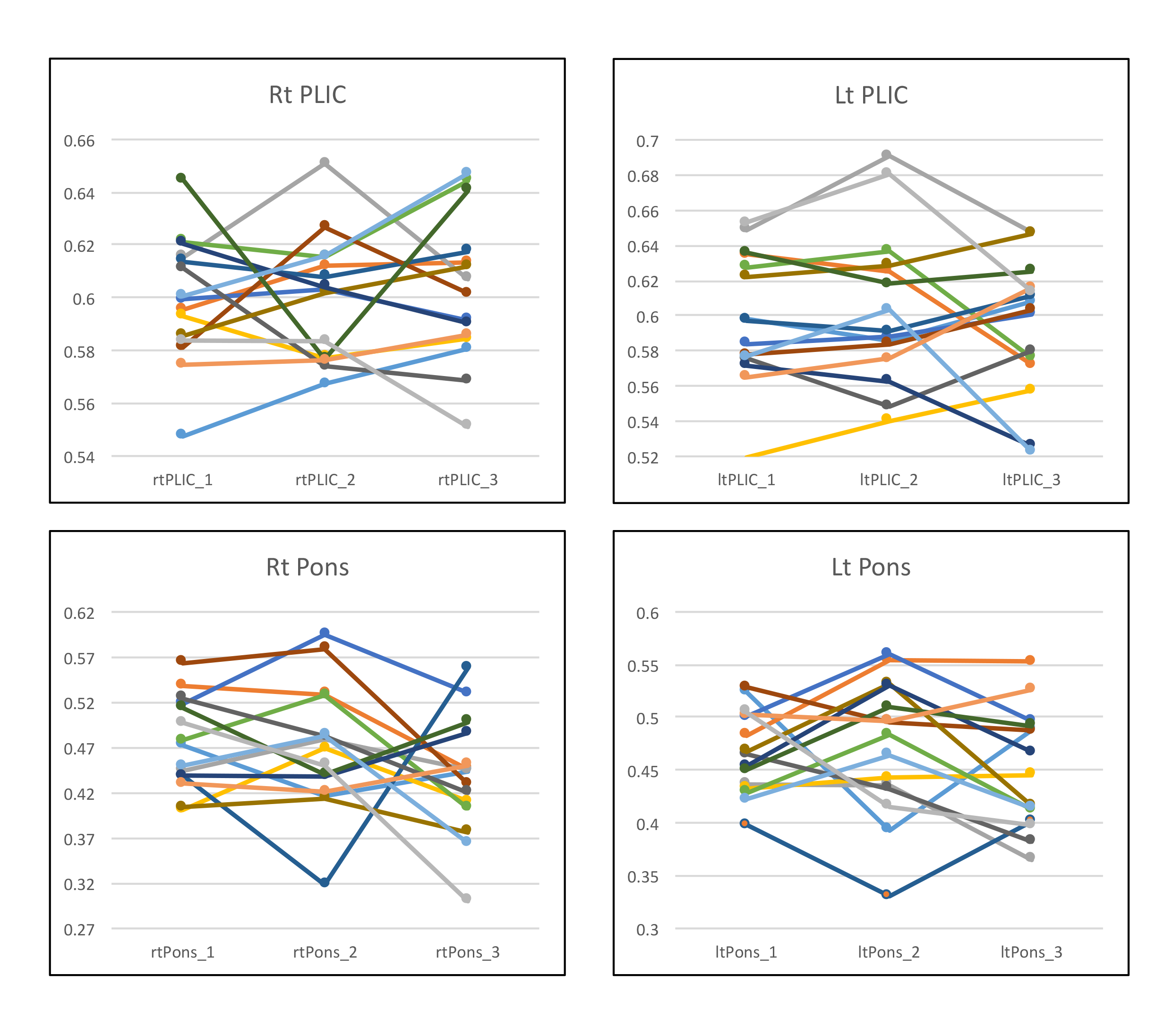

Supplement: Supplementary file 2 — Additional file 2 Figure S2. Manual ROI FA measurement for individual patients at three time-points for the CST at the pons and PLIC. Using MANOVA there were no significant difference among the mean values at each time-points. [file 12880_2020_489_MOESM2_ESM.tif]
