# Supplementary material for: Serial MRI studies over 12 months using manual and atlas-based region of interest in patients with amyotrophic lateral sclerosis
Source: BMC Med Imaging. 2020 Aug 3;20:90. doi: 10.1186/s12880-020-00489-w (PMC7397614; doi:10.1186/s12880-020-00489-w)
Supplement: Supplementary file 4 — Additional file 4 Table S1. Means (SD) for FA at three Time-points using atlas-based ROI and manual ROI. None of the regions, PLIC and CST at pons, showed significant differences overtime. [file 12880_2020_489_MOESM4_ESM.docx]

**Supp Table 1: Means (SD) for FA at three Time-points using atlas-based ROI and manual ROI.** None of the regions, PLIC and CST at pons, showed significant differences overtime.

| *Atlas based ROI* | | | | |
| --- | --- | --- | --- | --- |
| Region | FA1 mean (SD) | FA2 mean (SD) | FA3 mean (SD) | P value  (MANOVA) |
| rt CST | 0.462 (0.026) | 0.465 (0.042) | 0.465 (0.034) | 0.832 |
| lt CST | 0.456 (0.027) | 0.461 (0.035) | 0.459 (0.027) | 0.475 |
| rt PLIC | 0.559 (0.016) | 0.560 (0.020_ | 0.561 (0.019) | 0.690 |
| lt PLIC | 0.558 (0.021) | 0.561 (0.023) | 0.558 (0.025) | 0.644 |
| *Manual ROI* | | | | |
| rt CST | 0.474 (0.065) | 0.469 (0.070) | 0.437 (0.050) | 0.226 |
| lt CST | 0.466 (0.056) | 0.472 (0.064) | 0.450 (0.040) | 0.517 |
| rt PLIC | 0.599 (0.028 | 0.599 (0.023) | 0.602 (0.024) | 0.928 |
| lt PLIC | 0.599 (0.038) | 0.604 (0.044) | 0.594 (0.037) | 0.782 |
