# Supplementary material for: Serial MRI studies over 12 months using manual and atlas-based region of interest in patients with amyotrophic lateral sclerosis
Source: BMC Med Imaging. 2020 Aug 3;20:90. doi: 10.1186/s12880-020-00489-w (PMC7397614; doi:10.1186/s12880-020-00489-w)
Supplement: Supplementary file 5 — Additional file 5 Table S2. FA averaged mean of ROIs in both methods. [file 12880_2020_489_MOESM5_ESM.docx]

**Supp Table 2: FA averaged mean of ROIs in both methods.**

| ROI | Mean FA | SD |
| --- | --- | --- |
| Manual PLIC | 0.60 | 0.001 |
| Automated PLIC | 0.56 | 0.003 |
| Manual CST | 0.46 | 0.001 |
| Automated CST | 0.46 | 0.003 |
