# Supplementary material for: Serial MRI studies over 12 months using manual and atlas-based region of interest in patients with amyotrophic lateral sclerosis
Source: BMC Med Imaging. 2020 Aug 3;20:90. doi: 10.1186/s12880-020-00489-w (PMC7397614; doi:10.1186/s12880-020-00489-w)
Supplement: Supplementary file 6 — Additional file 6 Table S3. Correlation of FA in both methods. Left PLIC showed significant correlation using both ROI methods. [file 12880_2020_489_MOESM6_ESM.docx]

**Supp Table 3: Correlation of FA in both methods.** Left PLIC showed significant correlation using both ROI methods.

| ROI | p value | R value |
| --- | --- | --- |
| Rt PLIC | 0.069 | 0.482 |
| Lt PLIC | **0.011** | 0.636 |
| rt CST | 0.713 | 0.104 |
| lt CST | 0.296 | 0.289 |
